# Twenty-first century macro-trends in the institutional fabric of science: bibliometric monitoring and analysis

**DOI:** 10.1007/s11192-016-2041-z

**Published:** 2016-07-02

**Authors:** Robert J. W. Tijssen, Jos Winnink

**Affiliations:** 1 Centre for Science and Technology Studies (CWTS), Leiden University, The Hague, The Netherlands; 2 DST-NRF Center or Excellence in Scientometrics and Science, Technology and Innovation Policy (SciSTIP), Stellenbosch University, Stellenbosch, South Africa; 3 Dual PhD Centre, Leiden University, The Hague, The Netherlands; 4 Netherlands Enterprise Agency, The Hague, The Netherlands

**Keywords:** Basic science, Applied science, Research orientation, Bibliometric analysis, Web of science, 00A99, O3

## Abstract

Some say that world science has become more ‘applied’, or at least more ‘application-oriented’, in recent years. Replacing the ill-defined distinction between ‘basic research’ and ‘applied research’, we introduce ‘research application orientation’ domains as an alternative conceptual and analytical framework for examining research output growth patterns. To distinguish possible developmental trajectories we define three institutional domains: ‘university’, ‘industry’, ‘hospitals’. Our macro-level bibliometric analysis takes a closer look at general trends within and across some 750 of the world’s largest research-intensive universities. To correct for database changes, our time-series analysis was applied to both a fixed journal set (same research journals and conference proceedings over time) and a dynamic journal set (changing set of publication outlets). We find that output growth in the ‘hospital research orientation’ has significantly outpaced the other two application domains, especially since 2006/2007. This happened mainly because of the introduction of new publication outlets in the WoS, but also partially because some universities—especially in China—seem to have become more visible in this domain. Our analytical approach needs further broadening and deepening to provide a more definitive answer whether hospitals and the medical sector are becoming increasingly dominant as a domain of scientific knowledge production and an environment for research applications.

## Introduction

Both ‘basic research’ and ‘applied research’ usually have highly ambiguous and politicized meanings—either within the setting of science governance debates, as components in research funding models, or as pre-defined strategic research management objectives. Both concepts are often used imprecisely and may take on a range of connotations. ‘Basic research’ entered the world of science statistics in the mid-twentieth century, but scientists and science policy makers have questioned this concept ever since and disagree about what it actually constitutes (Godin 2003). Commonly used definitions of ‘basic research’ include: “… experimental or theoretical work undertaken primarily to acquire new knowledge of the underlying foundations of phenomena and observable facts, without any particular application or use in view” (OECD 2002), and “… as systematic study directed toward fuller knowledge or understanding of the fundamental aspects of phenomena and of observable facts without specific applications towards processes or products in mind” (OMB 2009).

The OECD definition was operationalized by statistical offices in some countries, but has never been generally adopted or implemented in all OECD-zone countries—thus hampering any kind of large scale statistical assessment of scientific research in terms of curiosity-driven, discovery-oriented ‘basic research’ or other ‘application-oriented’ types of scientific research. Attempts to develop alternative concepts, definitions and analytical frameworks have failed so far (see Calvert and Martin 2001 for an overview). Surveys among university researchers, to classify themselves in terms of their type of research, underscore the conceptual difficulties and the lack of applicability in real-life academic research settings (Calvert and Martin 2001; Gulbrandsen and Kyvik 2010; Bentley et al. 2015).

OECD definitions of ‘basic’ scientific research, as well as various types of application-centric research, lack analytical power to describe general patterns and macro-level trends within science. This gap in our understanding is unfortunate, particularly in this a day and age where growing numbers of universities are under increasing pressure to become more ‘mission-driven’ (rather than researcher-driven) to engage in societal challenges and provide economic value. We lack systemic information to gauge if and how these new ‘societal contracts’ are impacting on the way scientific research activity has evolved in recent years, especially in terms of the degree research has become more application oriented outside the academic domain.

In this study we examine the distribution of research publication output across institutional sectors, where we distinguish the university sector, the business sector, and the hospital sector. Where the university sector is seen as a ‘knowledge production environment’, the latter two sectors are typically a ‘knowledge application environment’. Our analytical approach builds on earlier related work by Tijssen (2010), who analyzed macro-level trends in the world’s scientific publication output, during the years 1999–2008, within Thomson Reuters’ *Web of Science* *Core Collection* database (from here on referred to by the acronym *WoS*). In this follow-up study, we monitored macro-level developments from 2000 to 2013 across the entire WoS, as well as within the world’s largest research universities. Our primary research questions were:

- Are there discernible general patterns and trends in worldwide science that suggest an ongoing or novel shift towards application-oriented science?
- If so, what are the possible determinants, and can one discern differences between research-intensive universities in their ‘application orientation’?

## Conceptual framework

Large research-intensive universities are among the key change agents in knowledge-intensive societies (Rauhvargers 2011). These organizations create new knowledge, technologies and human resources for societies and economies, contributing and adapting to national policies and regulations, while simultaneously reacting to international drivers and incentives with regards to worldwide competition and cooperation in modern-day science. Each university has a unique ‘institutional profile’ in terms of its disciplinary diversity and the distribution of the types of research done. For most research-intensive universities, being slowly evolving organizational entities, one expects little annual change within their research profiles, but identifiable and measureable shifts may occur over the course of a decade or longer. The emergence of world university rankings, which took off about 10 years ago, has become an increasingly important driver of institutional change and strategic positioning of research universities National or regional policy initiatives may also exert an impact on how universities re-orientate their research portfolio’s, for example to better align them to ‘smart specialization’ strategies of European regions and associated needs for universities to engage in application-centric research of local relevance (Foray 2015). How research universities respond to such externalities will differ; some are more inclined to adapt and adjust than others. Some might opt for unique ‘niche-driven’ development paths, others might pursue catching-up strategies similar to leading universities in their home country or elsewhere. The world’s largest comprehensive universities—often leading ‘global’ universities—are probably less willing to change drastically within a relatively brief period of time, because of the benefits that accrue from their global branding and reputation, and because of their economies of scale and scope that enable sustainable high-level performance (Mohrman et al. 2008).

The previous 20 years have introduced at least two high-profile theoretical models of scientific knowledge production that enable us take a broader view of its responsiveness to societal change: the Mode 2 model (Gibbons et al. 1994), and the Quadrant model (Stokes 1997). Both models are based on notions of interactivity between knowledge production within science-centric organizations and knowledge usage in external environments. Gibbons and colleagues assert that university-centric ‘Mode 1’ knowledge production spaces, with their traditional emphasis on basic research, will gradually be replaced by more heterogeneous ‘Mode 2’ institutional structures with a larger context-driven variety of knowledge production streams (Gibbons et al. 1994). Mode 2 science is “socially distributed, application-oriented problem-focused, trans-disciplinary and subject to multiple accountabilities” (Nowotny et al. 2003). Mode 2 includes not only the practice of application-centric research in universities, and collaboration across institutional sectors, but also the generation of research-based knowledge elsewhere in society. According to these authors, universities will contribute increasingly smaller shares in knowledge production, whereas research institutes, hospitals, think tanks, and other institutions will become more prominent (Gibbons et al. 1994, p. 85).

Clearly, science is not the exclusive domain of universities and public research institutes. Some for-profit business enterprises (‘industry’), and general hospitals and medical clinics (‘hospitals’) are also active in discovery research^1^—sometimes stand-alone, but usually in collaboration with universities or public research institutes. However, the business sector and hospital sector are much more inclined to absorb and apply university R&D findings (e.g. cures for diseases, medical devices) rather than to conduct in-house basic research. Contrary to academic hospitals, which tend to actively engage in basic research, general hospitals and clinics are more likely to perform application-oriented clinical research and focused on the medical practice (Hicks and Katz 1996; Gulbrandsen et al. 2016). Industry’s research is done in order to solve technical or marketing problems, where basic research (dome either within or outside the firm) tends to occur only in those promising areas where business enterprises see opportunities to get a return on investment in the long run (e.g. Rosenberg and Nelson 1994; Tornquist and Hoenack 1996). R&D-intensive business enterprises may outsource research, or cooperate extensively with universities or other public research institutes, in areas and topics with a good chance of appropriating enough of the outcomes to justify their investment.

## Research methodology

Our analytical framework is based on a research output perspective, i.e. that of research findings published in international peer-reviewed scholarly publication outlets. This approach fits within a research tradition of large-scale empirical studies that examine relationships and interactions between university science and associated knowledge application domains (Narin et al. 1976; Hicks and Katz 1996; McMillan et al. 2000; Godin and Gingras 2000; Boyack et al. 2014). Adopting such a perspective implies a focus on scientific knowledge creation, rather than on its possible applications, and accepting a restricted analytical scope where our information source reflects outputs of successful knowledge production processes that were accepted by reviewers for publication in peer-reviewed scholarly outlets.

Deriving our bibliographical data from the *Web of Science Core Collection* (WoS), we assume that WoS-indexed outlets (mainly scientific and technical journals) with a relatively large share of non-university authors, from either industry or the hospital sector, partially represent knowledge application domains in which those researchers are actively engaged with scientific research. Such ‘application-oriented’ outlets are more likely to include the results of research activities conducted in ‘Mode 2’ knowledge production environments. Publications of university-based researchers in those same outlets are, on the whole, also assumed to be more oriented toward ‘research areas’ (topics, issues and problems) that are still typical or representative for those application domains. These outlets may of course include still a significant share of discovery-oriented publications based on curiosity-driven research. In contrast, those outlets that have much higher shares of university-affiliated authors are assumed reflect areas of a more general ‘academic’ interest and further removed from (the potential for) practical applications outside academia.

Our operational definition of those application domains builds on the concept of ‘Journal Application Domain’ (JAD) as described in Tijssen (2010). In this follow-up study we merged the six JADs into the following three mutually exclusive ‘Research Application Domains’ (RADs):

- University research orientation (URO).
  WoS indexed outlets with a large share of research papers produced by university-affiliated researchers (excluding medical schools and university-affiliated health systems), i.e. <3 % of the author affiliate addresses refer to private sector business enterprises (‘industry’, excluding private higher education and private hospitals), and <3 % are located in the hospital sector.
- Industrial research orientation (IRO).
  WoS indexed outlets with a substantial share of papers (co-)produced by ‘industry’, i.e. more than 3 % of the authors are affiliated with the business enterprises, while <3 % are hospital affiliated.
- Hospital research orientation (HRO).
  WoS indexed outlets with a substantial share of papers (co-)produced by staff at non-academic ‘general’ hospitals, medical centers and clinics, i.e. more than a 3 % author share from hospital sector, and <3 % from industry.

The JAD classification scheme also includes a set of ‘industry–clinical relevant’ outlet assigned to both IRO and HRO (pharmaceuticals-related research mainly). These outlets were removed from our analysis rather than assigning them to both RADs—either on an equal basis or another (arbitrary) decision rule. These dual-orientation journals, almost 300 in total, account for about 7 % of all research publications in the WoS (see Tijssen 2010).

Introducing these three ‘research orientation’ domains shifts the analytical focus away from the intrinsic nature of research activities to the institutional environment(s) in which the research is done. If the growth of publication output in WoS-indexed HRO or IRO outlets outpaces the growth rate in URO outlets we assume that science is moving from university-dominated ‘basic research’ towards ‘application oriented’ where non-university sectors are more active. We measure the extent of these developments by counting the annual numbers of publications within outlets assigned to either URO, IRO or HRO. We applied this approach to the entire CWTS in-house WoS database, restricting our data collection to document types ‘research articles’ and ‘review articles’.

Our analysis shows a gradual increase in WoS indexed publication outlets from 2000 to 2010, after which it levels off at about 12,600.^2^ The publication output share of the medical, health and life sciences remains at 48–49 % since 2000, while the natural sciences and engineering sciences accounts for a stable 45–47 % of the total WoS indexed output.^3^ However, the new millennium has ushered in a major increase in the number of author affiliate addresses that CWTS has flagged as a ‘general hospital’, up from 68,000 in 2000 to 113,000 in 2013, whereas the number of addresses related to for-profit business enterprises (‘industry’) fluctuated between 71,000 in 2001 and 93,000 in 2011.^4^

Any study of such structural trends within the WoS needs baseline measurements to control for major database changes. We therefore defined two ‘source sets’ of selected publication outlets:

- A ‘fixed’ source set comprising of WoS indexed 6670 outlets, similarly to the preceding study, where each outlet must have published annually during the period 2000–2010. Improving upon that study, we now impose a second selection criteria in order to enhance the validity of our source set and statistical robustness of our data: at least 50 % of the author affiliate addresses have to assigned to a CWTS-imposed institutional sector, such as ‘university sector’, ‘business sector’ and ‘hospital sector’. The fixed source set represents a stable publishing environment and a unified analytical framework for time-series analysis, even though some outlets may change significantly in size and subject matter over a decade or more.
- A ‘dynamic’ source set, which also includes all new outlets that have entered the WoS since 2000 and outlets that were not continuously indexed. Newly added outlets reflect changes in the source coverage of world science, especially with regards to novel areas of research specialization (e.g. newly launched interdisciplinary outlets) or outlets that used to cover topics on the fringe of mainstream science (e.g. outlets from developing countries or regions). Each of the 13,485 outlets in this set complies with the 50 % criterion. The dynamic set is more likely to reveal emerging RAD trends in world science. University publication output in this source set however may represent various attribute of knowledge production processes: (a) output in new areas of science; (b) enhanced publication productivity in existing areas, but now published in new, WoS-indexed outlets, or (c) existing knowledge production that was not previously WoS-covered.

Rather than analyzing the entire publication output from all research-active universities, which is not a well-defined institutional sector, our study focuses on general trends and patterns within world’s 750 largest research universities. The bibliometric information at CWTS on the WoS indexed research publication outputs of these universities, each included in the 2014 edition of the *Leiden Ranking*, is of sufficiently high level to enable in-depth analysis. We assume that the information derived from this subset of research-intensive universities is fairly representative for the situation across all research universities worldwide.

## Results

### General trends in research orientation

**Fig. 1:**
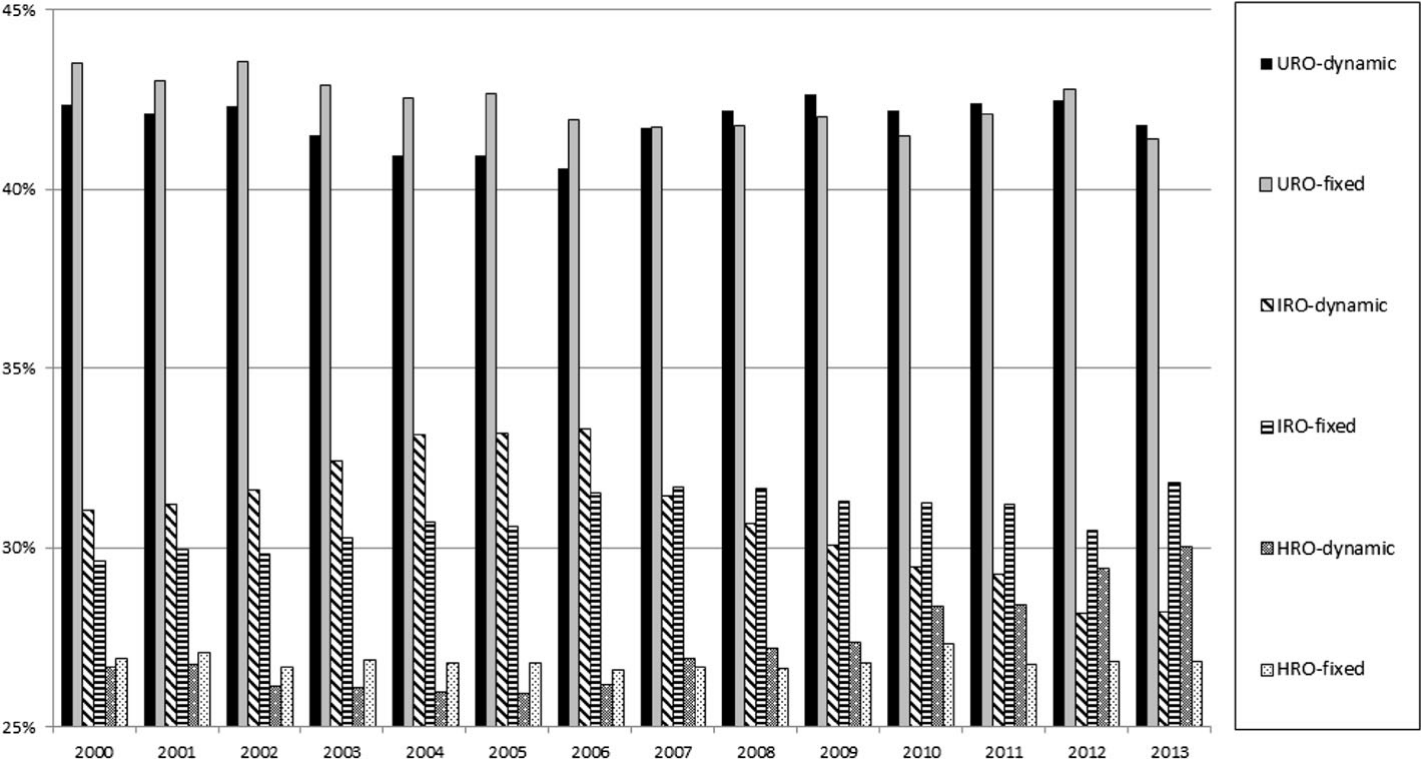
Share of Research Application Domains in world academic science (2000–2013, 750 universities)

Focusing on the longitudinal trends within 750 universities, Fig. 1 exhibits the changes in RAD distributions within the dynamic set and fixed set. The dynamic set shows URO shares moving between 40 and 43 % of the total publication output. IRO slowly declines from 31 to 28 %, whereas HRO moves up gradually from 27 to 30 %. As to be expected, the trends in the fixed set are less volatile, where URO, IRO and HRO shares each fluctuate within a 2–3 % bandwidth.

**Fig. 2:**
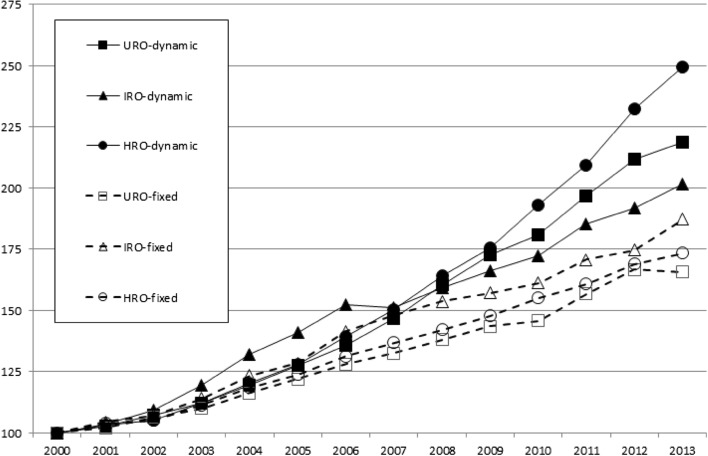
Growth patterns in research orientation within world science (2000–2013, 742 universities). Eight of the 750 universities we deleted due to low publication output volumes in either HRO or IRO domains and associated unreliable growth rates

Figure 2 depicts the growth rates of each RAD. Overall, we find a doubling of output, which reflect the expansion of the WoS since 2000. The dynamic set shows the largest growth over time, especially science 2006/2007. In more recent years HRO-dynamic growth rate seems to be accelerating and clearly outpacing both URO and IRO growth.

If science has become more ‘application oriented’ it is more likely to have occurred in terms of HRO. Our in-depth analysis will therefore focus on HRO trends, guided by the key research question: are changes in HRO-dynamic predominantly or exclusively induced by the ‘exogenous’ overall expansion of the WoS database, or are there possibly other ‘endogenous’ determinants?

### The rise of hospital research orientation

The WoS expansion is indeed a contributing factor. The 2006/2007 discontinuity is indeed caused by changes in the WoS coverage of the world’s scholarly literature, including the launch of journal *PLoS One* in December 2006, which has undergone a major expansion in terms of publication output. *PLoS One* had a significant impact on HRO growth patterns since 2006/2007, where this journal alone accounts for 7.5 % of all publications in the HRO domain and for 17.0 % of the HRO growth. *PLoS One* is now the world’s largest journal by number of papers published (about 30,000 per year). It’s family members, *PLoS Medicine* and *PLoS Clinical Trials*, are also HRO-assigned journals.

The observed surge in HRO-related publication output growth results from funding hikes for biomedical and clinical research. A recently published study, on US and international funding of medical research, on suggests as much (Moses et al. 2015): the findings indicate a 6 % annual increase in total US funding between 1994 and 2004, followed by a declined down to .8 % per year in the period 2004–2012. Given the fact that the USA accounts for some 30 % of the WoS indexed publications, such increases are bound to affect the size of the WoS. China, according to the same study, tripled its funding between 2004 and 2012, which should also exert a major impact on the WoS coverage of the medical research literature.

Collectively, the increased funding of medical research in these major countries seems to be a major contributor to the growth of HRO-dynamic. But the levels of HRO-fixed have hardly moved, which would imply that this surge of medical research funding helped to launch many new WoS indexed research journals to accommodate this publication influx.

The bias created by the introduction and rise of the *PloS One* and its family members in the WoS requires a separate time-series analysis for the years 2007–2013. If more HRO-journals have entered the WoS since 2006, has this changed the composition of those journals in terms of research specialization? Some newcomers may focus on discovery-oriented (‘biomedical’) research, others on application-oriented (‘clinical’) research. If we can discern a rise of publications in journals that belong to the latter category, this would suggest that at least the medical research segment of world science is becoming more application-oriented. To examine this issue, HRO-dynamic and HRO-fixed were both divided into two mutually-exclusive subgroups:

- ‘HRO-low/medium intensity’: publication outlets with 3–20 % share of papers that are (co-)produced by staff at general hospitals, medical centers and clinics.
- ‘HRO-high intensity’: outlets with a share above 20 %. These outlets contain many publications, with hospital affiliations, on clinical research and medical practice applications.

**Fig. 3:**
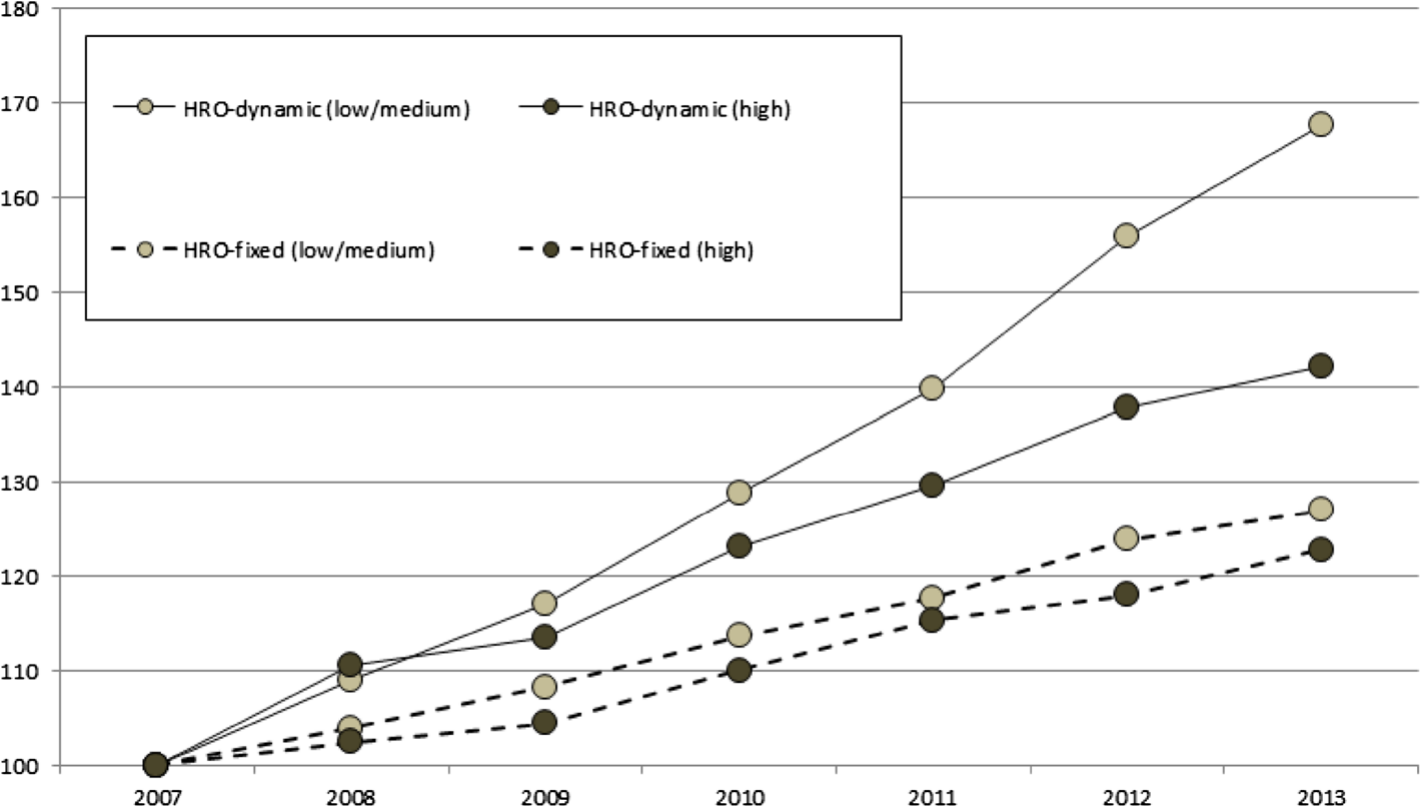
HRO growth patterns by HRO subgroup (2007–2013, 742 universities)

If university research, especially in the Medical, Health and Life sciences (MHL sciences), is truly moving towards a larger focus on applications (such as high-tech clinical care and science-based medical technologies), one expects to find significant growth in the HRO-high subgroup. Figure 3 shows the growth rates of both HRO subgroups in terms of publication output, the fixed source set providing baseline information.^5^ We observe significant growth in both, but the low/medium subgroup is growing at a much faster pace—this trend does not occur in the fixed source set. HRO-low/medium subgroup accounted for 26.2 % of the total WoS publication output in 2013, having gradually increased to that level since 2007, while the HRO-high share remained fairly constant at 1.7 %. Nonetheless, the 40 % rate of the HRO-high subgroup (the equivalent of a 6 % Compound Annual Growth Rates—CAGR) signifies a noticeable increase of publication outlets with extensive contributions from hospital-based researchers. This trend may signify a small but gradual shift towards research of relevance for clinical practice in hospital settings.

Further analysis of the universities contributing to this trend reveals an interesting outcome: China is a major contributor in the group of universities with CAGR > 10 % in HRO-high (see Table 1). If we impose a lower threshold of 25 publications in 2013 in the outlets assigned to HRO–high, we find a small group of 16 very active universities, six of which are Chinese. How significant is this ‘China effect’? We expand our scope to the larger subset of universities with significant HRO-dynamic increases during the period 2007–2013. Here we find 32 universities where the HRO publication output increased by a CAGR of 20 % or more. A major share of those universities are Chinese, along with some from the Middle East and other countries in East Asia. Some of these Chinese universities in the ‘fast track’ group are catching up in the MHL domain, notably: China Medical University, Huazhong Agricultural University, and Nanjing Agricultural University. Others seem to be shifting towards HRO and MHL sciences, show a strong focus on the Natural Sciences and Engineering (NE sciences). Many of those universities have started from a low baseline (i.e. a few WoS indexed MHL publications), which explains their relatively high growth rates in HRO. The Chinese universities with NE research specialization rates of more than 90 % are: Northwestern Polytechnical University and the National University of Defense Technology.

**Table 1:** Summary description of high-growth HRO university subgroups (2007–2013, 742 universities)

	HRO-high (CAGR > 10 %)	HRO (CAGR > 20 %)
HRO rate (HRO share of total publication output) (%)	37	6
Specialization in MHL sciences (share of total publications) (%)	29	25
Specialization in NE sciences (share of total publications) (%)	16	60
Total number of universities	16	32
Number of Chinese universities	6	17

Given the relatively low number of publications involved, one cannot rule out the possibility that these distinctive patterns are caused by changes in publications habits and practices at these universities. More specifically, shifting from MHL publication output in (non-WoS) local-language journals to disseminating research findings in English-language WoS indexed outlets.

### Possible determinants of hospital research orientation

China and several other Asian countries are among the world’s high-profile emerging economies, nations that are moving from an applied, demand-led R&D base to more advanced science-innovation systems. Many research universities in these countries are in a process of structural change to become more effective and competitive internationally (e.g. Deema et al. 2008). These changes are diverse and may include: new research strategies and policies, new research facilities, collaboration with local hospitals, more activity in clinical trials, expanding networks of research partners, shifts in research specialization profiles, international publication strategies, improved research publication productivity, revised performance assessment systems. Many of those organizational features are university specific, and only a few can be reliably measured in an internationally comparative manner.

Can those selected metrics shed further light on organizational factors that may have contributed to those HRO growth rates in recent years. What are the general patterns, complement to those described in Table 1 with regards to the high-growth universities? To address this question, we computed CAGRs for all 742 universities to measure changes in HRO-dynamic during the years 2007–2013. Our linear regression model^6^ uses a size-corrected measure as a dependent variable: the CAGR of the share of the university’s total publication output within HRO-dynamic outlets. The following seven independent ‘predictor’ variables were selected from available data sources, describing those features of a university’s scientific research profile (size, specialization, and impact) that we were able to collect systematically for all selected universities:

- HRO rate—% of publication output in ‘hospital research orientation’ outlets (2013).
- IRO rate—% of research output in ‘industry research orientation’ outlets (2013).
- Research specialization MHL—share of research output in Medical, Health and Life sciences (data source: *Leiden Ranking 2014*, publication years 2009–2012).
- Research specialization NE—share of research output in Natural sciences and Engineering sciences (data source: *Leiden Ranking 2014*, publication years 2009–2012).
- Research output size—total research publication output (data source: *Leiden Ranking 2014*, publication years 2009–2012).
- Research interdisciplinarity—interdisciplinary research score (data source: *U*-*Multirank*, publication years 2009–2012).
- Research impact—share of publication publications among the Top 10 % most highly cited publications worldwide per field (data source: *Leiden Ranking 2014*, publication years 2009–2012).

Table 2 describes the outcome of the regression analysis, where four variables show significant contributions, all related to a university’s research specialization profile. The university’s research size, its international scientific impact, or its level of interdisciplinary research appear to be much less relevant. Interestingly, HRO growth rates correlate both with high levels of HRO publication outputs (and associated MHL specialization) and/or IRO (and NE specialization). In other words, the universities with high HRO growth rates have relatively low HRO orientation or have specialized research profiles, either in MHL and/or NE. This finding suggests the presence of catching-up processes within MHL-dominated universities (with medical schools and university hospital), or cases where universities are gradually shifting from NE-dominated profiles to MHL profiles.

**Table 2:** Results of regression analysis of CAGR of share of HRO-dynamic publications (2007–2013, 742 universities)

	Standardized coefficients		
	*β*	*T* value	Significance
HRO rate	−.12	−1.93	.06
IRO rate	.35	5.98	.00
Research specialization—MHL	.14	1.82	.07
Research specialization—NE	.19	2.44	.02
Research output size	−.02	−.46	.64
Research impact	−.00	−.08	.94
Research interdisciplinarity	.03	.80	.43
Constant		−1.59	.11
*R* ^2^ (variance explained)	.25		

However, the regression model explains only 25 % of the variance in the data. More empirical information is needed for a robust statistical analysis. Many other possible determinants affecting university HRO patterns and trends will remain unexamined because of data scarcity. Notable, because of missing data on joint affiliations of academic researchers with general hospitals, national policy initiatives for public health research, influx of funding for medical research, changes in clinical trials practices, and R&D linkages of pharmaceutical industry. At this point one cannot rule out the possible hidden effects of other (minor) determinants, or indeed slight shifts towards more application-centric research.

## General discussion and observations

In this macro-level empirical study we examined changes in publication output distribution patterns, especially during the years 2007–2013. By introducing an operational alternative to the conceptually ambiguous distinction between ‘basic research’ and ‘applied research’, we conducted a trend analysis of three ‘research application orientation’ domains in search of macro-level changes in the institutional orientation of world science: ‘university research orientation’ (URO), ‘industry research orientation’ (IRO) and ‘hospital research orientation’ (HRO).

We find signs of a possible structural change in favor of HRO, which may imply an institutional shift towards general hospitals and clinics as an scientific knowledge production environment. Results of our statistical analyses suggest this is predominantly caused by the interplay between: (1) increase of medical research outlets in the *Web of Science* database; (2) increased publication output by universities located China and other developing national science systems. Judging by our results the upsurge is more likely to have resulted from the compound effect of WoS coverage expansions, more funds for medical research, changing research specialization profiles, and changes in research publication practices and incentive systems in China and other emerging economies. Although there is no clear indication that science has become more application-oriented, the slight increase of publication output in HRO-intensive outlets during the last years, with many publications (co-)produced by staff at non-academic hospitals and clinics, may reflect a subtle and gradual creep towards a stronger focus on clinical research medical applications (Matthews 2015).

This study documents the change of journal composition in WoS, but cannot make convincing claims about the change in science overall, without further studies. It is too early to say if these observed changes mark a transition process towards a knowledge creation system with an increasingly large share of HRO related science. More research is needed with more sophisticated methods and appropriate databases, especially at the meso-level and micro-level. We need to extend our scope beyond the world’s top 750 research-intensive universities, which may have produced a misleading impression of trends throughout university research worldwide. Our current analytical model is also too crude in the sense that it classifies publication outlets in their entirety to application domains: all research publications in the thousands of outlets we examined (either a journal or conference proceedings) are simply assumed to be comparable in terms of their research orientation.

Moreover, general hospitals might well be playing a more important role in science, but whether hospital-based research is more application oriented remains an open question. A more fine-grained classification system is needed, at the level of individual publications, to systematically examine this more closely. Such micro-level case studies should disentangle possible trends and patterns by type of performer and by the type of performed science: some hospital-affiliated researchers, or their research programs, might actually be more active in discovery research (i.e. URO) than in application oriented research (i.e. HRO). Validation studies could be also address meso-level questions at the level of individual universities or hospitals (e.g. have major institutional changes has an impact?) or at the macro-level of entire national university systems (e.g. are government policies taking effect?).^7^

In conclusion, our RAD-based analytical framework and current bibliographic database does not allow us to control adequately for, or eliminate, all major biases and confounding factors that could help us either confirm or refute the notion that science has become more application-oriented. For now, it seems that the HRO domain is the main candidate to seek further information. However, additional data sources are sorely missed, and further research is required to unearth the missing variables (especially with regards to changes in IRO-related research output). One of those hidden factors could be sectoral differences in incentive systems for publishing research findings in the open scientific literature, where hospital researchers are encouraged to publish while industry’s knowledge appropriation and intellectual property protection regimes may have a detrimental effect on IRO-related publication output growth.

## Footnotes

1 We define ‘discovery research’ as curiosity-driven, discovery-oriented research with a relatively long time-horizon.

2 Sources qualify for this count if at least one publication (either a ‘research article’, ‘review article’, or a ‘letter’ document type) was indexed in the Web of Science.

3 The WoS-based operationalisation of these broad fields of science is identical to the one used for the CWTS *Leiden Ranking*.

4 The classification of author affiliate addresses to institutional sectors is done by a expert team within CWTS and focusses on the WoS-indexed publications of the world’s largest research universities.

5 A similar trend analysis of comparable IRO subgroups shows no such significant differences. The scores in 2013 are: IRO-dynamic (low/medium)—135; IRO-dynamic (high)—112; IRO-fixed (low/medium)—127; IRO-fixed (high)—120.

6 We opted for linear regression analysis because pair-wise curve fitting provided no convincing evidence, for any of these variables, of a better fit when using nonlinear relationships.

7 To enable such validation studies, CWTS is willing to share its URO/IRO/HRO coding system with external researchers to conduct small-scale validation studies. Please contact the authors for further information about access and usage conditions.
